# The Effect of a DNA Damaging Agent on Embryonic Cell Cycles of the Cnidarian *Hydractinia echinata*

**DOI:** 10.1371/journal.pone.0011760

**Published:** 2010-07-23

**Authors:** Tin Tin Su

**Affiliations:** Department of Molecular, Cellular and Developmental Biology, University of Colorado, Boulder, Colorado, United States of America; University of Texas MD Anderson Cancer Center, United States of America

## Abstract

The onset of gastrulation at the Mid-Blastula Transition can accompany profound changes in embryonic cell cycles including the introduction of gap phases and the transition from maternal to zygotic control. Studies in Xenopus and Drosophila embryos have also found that cell cycles respond to DNA damage differently before and after MBT (or its equivalent, MZT, in Drosophila). DNA checkpoints are absent in Xenopus cleavage cycles but are acquired during MBT. Drosophila cleavage nuclei enter an abortive mitosis in the presence of DNA damage whereas post-MZT cells delay the entry into mitosis. Despite attributes that render them workhorses of embryonic cell cycle studies, Xenopus and Drosophila are hardly representative of diverse animal forms that exist. To investigate developmental changes in DNA damage responses in a distant phylum, I studied the effect of an alkylating agent, Methyl Methanesulfonate (MMS), on embryos of *Hydractinia echinata*. Hydractinia embryos are found to differ from Xenopus embryos in the ability to respond to a DNA damaging agent in early cleavage but are similar to Xenopus and Drosophila embryos in acquiring stronger DNA damage responses and greater resistance to killing by MMS after the onset of gastrulation. This represents the first study of DNA damage responses in the phylum Cnidaria.

## Introduction

Damage to DNA, such as that caused by ionizing radiation or alkylating agents, elicits three well-studied responses in eukaryotes: cell cycle arrest by checkpoints, DNA repair and cell death [1]. The first two may be considered pro-survival and the last anti-survival, at least at the level of cells. How cells choose between these two fates remains to be fully understood. Understanding this decision may be of clinical importance, for example in directing cancer cells to death but normal cells to survival after radiation treatment. Preferential choice of survival over death or vice versa upon DNA damage appears to correlate with developmental stage in embryos of *Drosophila melanogaster* and *Xenopus laevis*.

Embryogenesis in Drosophila and Xenopus begins with an exponential increase in cell number that results from rapid cleavage of the large externally deposited egg. Concomitant with the onset of gastrulation, the increase in cell number slows as cell cycles lengthen. Cell cycle lengthening occurs via the introduction of a gap phase for the first time to the cell cycle, G1 in Xenopus and G2 in Drosophila [reviewed in [2]]. These changes in the very nature of the cell cycle accompany several changes in the embryo such as the start of transcription of many zygotic genes, and constitute the Mid-Blastula Transition (MBT) in Xenopus and Maternal to Zygotic Transition (MZT) in Drosophila. Longer interphases that follow MBT/MZT may allow cytoskeletal arrangements that are necessary for gastrulation but are incompatible with cell division [reviewed in [2]]. Rapid cleavages followed by longer cell cycles are seen in representatives of all major phyla examined that include chordata (frog), echinodermata (star fish and sea urchin), arthoropoda (fruitfly), annelidata (leeches), mollusca (clam), nematoda (*C. elegans*) and cnidaria (Hydractinia) [[3]; [4] and references therein].

Changes in cell cycle structure that occur during embryogenesis in Drosophila and Xenopus are also concomitant with an increased ability to regulate the cell cycle in response to DNA damage. In cleavage cycles of Drosophila, which occur in a common cytoplasm, nuclei with damaged or incompletely replicated DNA enter mitosis after a delay. For example, interphase in embryonic cycles 11 and 12 normally lasts about 10 min. Injection of the DNA polymerase inhibitor Aphidicolin in these cycles lengthens the interphase by an additional 10–15 min [5], [6]. Once the nuclei enter mitosis, however, DNA defects activate a Chk2-dependent checkpoint that inactivates the centrosome and disrupts mitotic spindle function [7], [8]. Consequently, chromosome segregation fails and the resulting polyploidy nuclei exit mitosis to be incorporated into the yolk mass. Thus there are clear active responses to DNA damage even in cleavage stages in Drosophila. In post-MZT cycles that include a G2 phase, treatment with similar doses of DNA damaging agents now delays the entry into mitosis via inhibitory phosphorylation of Cdk1 [9]. This is similar to the response in somatic cells [10]. In sum, syncytial cleavage cycles before MZT appear to favor abortive mitosis and culling of damaged nuclei; this response is not seen in cell cycles after MZT that appear to favor cell cycle arrest. Correlating with these changes in DNA damage responses before and after MZT are resistance to killing by DNA damaging agents; post-MZT embryos show higher resistance to killing by IR than pre-MZT embryos [[9], [11]; our unpublished data]. The increased resistance could, however, be due to other changes besides checkpoints such as the onset of a zygotic transcription program.

In contrast to Drosophila cleavage cycles, Xenopus cleavage cycles are found to lack DNA damage checkpoints. Checkpoints are activated in response to DNA damage after MBT, at which time cell cycles also acquire a gap phase. Injection of extra (undamaged) DNA into 2-cell stage embryos results in the ability of embryos to slow the cell cycle in response to DNA damage, leading to the conclusion that checkpoint activation becomes possible only after a certain DNA/cytoplasm ratio is reached during embryogenesis in this system [12], [13]. It has been argued that “relaxed” checkpoints in early embryos increase mutation rate and help accelerate evolution during environmentally stressful periods [14]. As in Drosophila, the resistance to killing by DNA damaging agents increases after MBT in Xenopus.

Xenopus and Drosophila, two of the best-studied models for embryonic cell cycle regulation, are hardly representative of all metazoan. Indeed, model organisms have been selected for ease of culture in artificial laboratory conditions and may show characteristics such as rapid life cycle and great reproductive success under a wide range of conditions that are absent in other metazoa. As such, regulation of cell proliferation under normal and adverse conditions in these organisms may or may not be representative of those in other metazoans. Given that exposure to DNA damaging agents is a universal experience of all living things, I asked if developmental changes in DNA damage responses described above for Drosophila and Xenopus are also conserved in *Hydractinia echinata*, a colonial hydroid and a member of the phylum Cnidaria. Cnidaria includes corals, sea anemones, jellyfish and hydra that show radial symmetry in body plan and only two germ layers separated by mesoglea, and is one of the most evolutionarily distant major metazoan phyla that still show true tissues [15], [16], [17] (Supplemental Figure S1). I find that DNA damage responses in Hydractinia show both similarities to and differences from those in Xenopus and Drosophila. I hope these studies will encourage the use of non-traditional animals in order to probe the range of basic cell biological phenomena such as the cell cycle and checkpoint regulation among metazoans.

## Methods

### Hydractinia culture

Hydractinia colonies on hermit crabs were obtained from the Aquatic Resources Division of the Marine Biology Laboratory, Woods Hole, MA, during the summer of 2008. The animals were placed in the dark for at least 4 hr prior to light-induced spawning. Spawning was detected visually by sperm and egg release. Embryos were collected using transfer pipettes and placed in filtered seawater (FSW). 2 and 4-cell stage embryos were manually selected for drug treatment. The room temperature was monitored to be 22±1°C.

### Fixation and antibody staining

Embryos were fixed by incubation in 3.7% formaldehyde in FSW for 30 min at room temperature. Fixed embryos were washed thrice in PBT and incubated in the primary antibody in PBT overnight at 4°C. Embryos were washed thrice in PBT for 5 min each and incubated in secondary antibodies for 3 hr at room temperature. To visualize DNA, embryos were stained with 10 µg/ml bisbenzimide (Molecular Probes) in PBT before mounting in Fluoromount-G. Primary antibodies were 1∶1000 rabbit polyclonal against phospho-Ser10-Histone H3 (Upstate Biotechnology) and 1∶100 monoclonal against β-tubulin (E7; Developmental Hybridoma Bank). Fluorescent secondary antibodies were used at 1∶500 in PBT.

### Image acquisition

**Figure 1:**
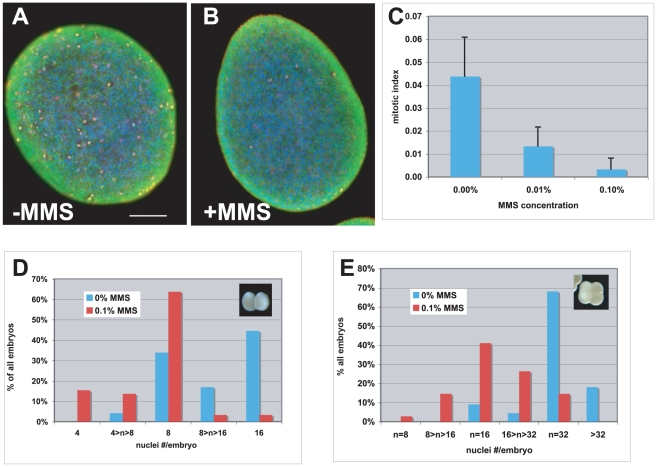
MMS inhibits mitosis in gastrula and cleavage stage embryos. (A, B) Gastrula stage embryos were incubated in filtered seawater (FSW) containing 0 (- MMS) or 0.1% (+ MMS) MMS for one hour before fixing and staining with antibodies to detect phospho-Histone H3 (red) and β-tubulin (green). The embryos are also stained with bisbenzamide to visualize DNA. (C) Mitotic index after 1-hour incubation in FSW containing various concentrations of MMS is quantified. The data are from 2690 cells in 11 embryos (0%), 3055 cells in 10 embryos (0.01%) and 2892 cells in 11 embryos (0.1%) in two different experiments. Error bars represent one standard deviation each. (D, E) Histograms show the percent of embryos that show nuclei number (n) per embryo as indicated when 2-cell (D) and 4-cell (E) stage embryos were incubated in 0 or 0.1% MMS in FSW for one hour, fixed and stained as in A and B. Mitotic figures from prophase to anaphase were counted as one nucleus each. Telophases (see Supplemental Figure S2) were counted as two nuclei each. Additional information on these data sets are in Table 1.

**Figure 2:**
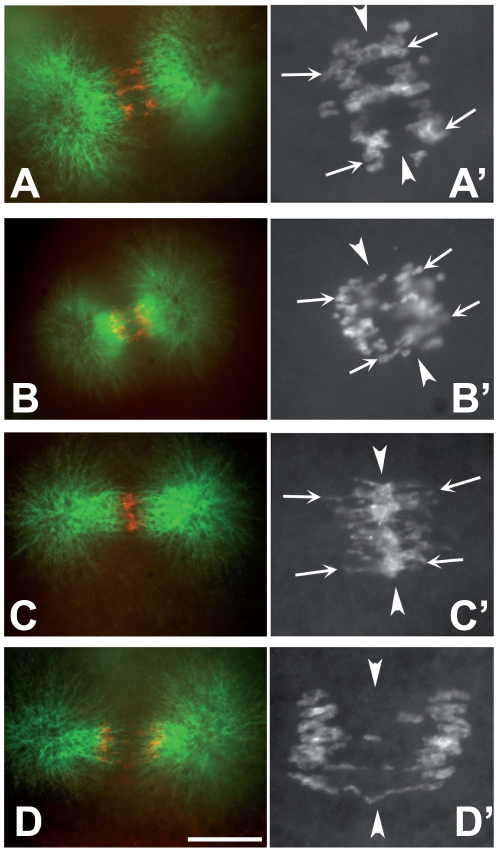
Chromosome segregation failure in the presence of MMS and caffeine. 4-cell stage embryos were incubated for one hour in FSW containing various drugs, fixed, and stained for PH3 (red) and β-tubulin (green). Anaphase figures are shown. Arrowheads indicate the metaphase plate and arrows indicate the leading edge of segregating chromosomes. (A, B) Control (A) and 0.1% MMS-treated (B) embryos show successful chromosome separation with little or no chromosome material remaining at the metaphase plate. PH3 signal is shown magnified in A' and B' respectively. (C, C') The presence of 5 mM caffeine in addition to MMS led to chromosome segregation failure. C' shows the PH3 signal after magnification. The extent of chromosome separation is similar to that in A' (compare leading edged), but most of the PH3 signal remains at the metaphase plate (arrowheads). (D, D') 5 mM caffeine alone allows successful chromosome separation. This figure is in later stage of anaphase then the preceding ones. Scale bar  = 10 µM in A-D, 4 µM in A'-D'.

**Figure 3:**
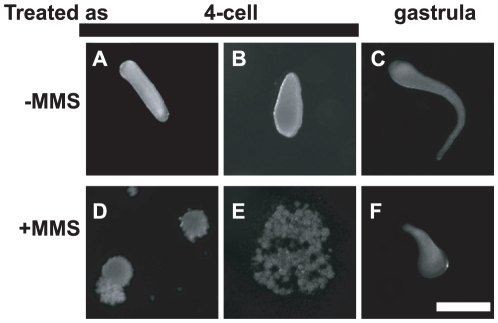
Survival after MMS treatment of cleavage and gastrula stage embryos. (A, B, D, E) Embryos in the 4-cell stage were treated with 0% (−) or 0.1% (+) MMS for one hour. Embryos were transferred to drug-free FSW and examined after 24 hr. All of the control 4-cell stage embryos survived as indicated by their ability to reach the gastrula and planula stages (A, B). Most drug-treated 4-cell embryos did not survive as indicated by signs of disintegration (D, E). (C, F) Gastrula stage embryos at 14–16 hr after the first sign of spawning were treated with MMS and their survival examined as above. All of the control embryos and most of the MMS-treated embryos survived as indicated by their ability to reach the swimming planula stage. Development appears slower after incubation in MMS, but this issue remains to be studied rigorously. Animals in C and F were of similar size but oriented differently. Scale bar  = 200 µM.

Fluorescence images were acquired on Nikon (Figure 1) and Zeiss (Supplemental Figure S2) compound microscopes attached to CCD cameras. The reason for the use of different microscopes was that I utilized resources from a 6-week summer Embryology course at MBL; microscopes available at any given time had to be the ones used. Images in Figure 2 were acquired on a Leica DMR microscope with a Sensicam CCD camera and Slidebook software (Intelligent Imagining Innovations) after my return to Boulder. Bright field images (Figure 3) were acquired using a CCD camera attached to a dissecting microscope and Spot imaging software (Diagnostic Instruments, Inc.). Images were saved as TIFF files, processed and assembled using Photoshop (Adobe Systems).

**Table 1:** Summary of results from six experiments.

expt. #	Starting stage	treatment	# embryo examined	Ave. doubling time (min)	Relative Mitotic index
1	2-cell	none	14	23.9	1.00
		0.1%MMS	13	32.9	0.65
2	2-cell	none	47	23.0	1.00
		0.1%MMS	58	31.4	0.32
3	4-cell	none	44	19.2	1.00
		0.1%MMS	49	28.6	0.09
4	4-cell	10 mM Caf.	7	102.6	N/A
5/6	4-cell	none	14	21.6	1.00
		0.1%MMS	20	32.1	0.34
		5 mM Caf.	12	32.0	1.67
		MMS+5 mM Caf.	18	34.2	0.67

### Data analysis

Nuclear doubling time (T in min) was calculated from the fold increase in nuclei number for the whole sample (F) during a 60 min incubation, using the formula T = 60/log_2_F where log_2_ = log base 2. Standard deviation of a population was calculated using the Student's t-test. Statistical significance of embryo survival was calculated using Fischer's Exact Test.

## Results

Cnidarian embryos generate only two germ layers, ectoderm and endoderm (called ‘entoderm’). Despite this simplicity, Cnidarian gastrulation is complex; all modes of gastrulation seen in embryos with three germ layers are also seen in Cnidarian embryos [18]. Embryogenesis in the cnidarian *Hydractinia echinata* (to be called Hydractinia hereafter) begins with cleavage divisions that result in an exponential increase in cell number until about the 400-cell stage (after∼9 doublings). Subsequent increase in cell number is slower and cell number plateaus at about 6,000 cells (after ∼4 more doublings) [3]. Interestingly gastrulation in Hydractinia begins at the 16-cell stage, during rapid cleavage and prior to the slowing down of the cell cycle [18]. Hydractinia gastrulation occurs by mixed delamination, a combination of directed cell division and multipolar ingression to internalize cells to form the presumptive entoderm.

To identify developmental changes in DNA damage responses in Hydractinia, I (a) assayed for changes in mitotic activity upon exposure to MMS before and after the start of gastrulation, and (b) measured the survival of embryos after MMS exposure at these times in development. MMS is a DNA alkylating agent that causes both single and double strand breaks. To study the effect of MMS on mitosis, I first confirmed that mitosis in Hydractinia could be detected using a combination of a DNA stain and antibodies against Xenopus β-tubulin and human phospho-Ser10 Histone H3 (pH), a mitotic marker (Supplemental Figure S2). These antibodies have not been characterized in Hydractinia previously, although the pH3 antibody has been used on at least one other Cnidarian [fresh water Hydra; [19]].

### MMS inhibits mitosis in Hydractinia embryos

Using these reagents, I find a MMS dose-dependent response in mitotic inhibition in 12–16 hr old embryos (i.e. 12–16 hr after spawning; Fig. 1 A–C). This stage was chosen to ensure that all embryos have begun gastrulation, which begins at the 16-cell stage as described above. Embryos in this stage have also completed the exponential increase in cell number due to rapid cleavages, which decelerate after about 9 divisions or at ∼400-cell stage as described above. 12–16 hr old embryos contain >2000 nuclei per embryo. Based on findings in Xenopus and Drosophila embryos, gastrulae would be expected to display at least some response to MMS. The range of concentration of MMS needed, 0.01–0.1%, is comparable to concentrations used in Drosophila, human cells and yeast. 0.1% MMS (9.1 mM) produced the strongest response, a 15-fold reduction in mitotic index, and was used to assay for mitotic inhibition in pre-gastrulation embryos (Fig. 1, D and E).

To examine the effect of MMS during pre-gastrula stages, I treated 2-cell and 4-cell stage embryos with MMS. I found that 0.1% MMS inhibited but did not completely block mitosis in 2-cell and 4-cell stage embryos (Figure 1D,E). 2-cell embryos incubated without MMS underwent 2 to 3 nuclear divisions in 1 hr to produce embryos with 8 to16 nuclei per embryo. 2-cell embryos incubated in MMS produced mostly 8-nuclei embryos. 4-cell embryos incubated without MMS also underwent 2 to 3 divisions in 1 hr to produce mostly 32-nuclei embryos. 4-cell embryos incubated in MMS produced mostly 16-nuclei embryos. The increase in doubling time was similar for both 2-cell and 4-cell embryos in four independent experiments (Table 1; Materials and Methods for calculation), from 21.9±2.0 min (n = 119) in controls to 31.2±1.9 min (n = 140) in MMS-treated embryos. The decrease in mitotic index varied widely from experiment to experiment but was on the average 3-fold (Table 1, expts. 1,2,3,5/6). These results indicate that cleavage divisions slowed by about 50% in the presence of MMS but continued nonetheless. The doubling time for untreated embryos reported here is less than the previously published value but this difference could be due to the fact that incubations were at 21°C in the present study and at 18°C in the previous study [3].

### Mitotic chromosome segregation can succeed in the presence of MMS unless caffeine is present

In Drosophila, cleavage stage mitoses that occur in the presence of DNA damaging agents show chromosome bridges and the complete failure to segregate chromosomes. The failure in chromosome segregation is an active, Chk2-dependent checkpoint response achieved by inactivation of centrosomes via the loss of γ-tubulin Ring Complex members from these structures and consequent loss of astral microtubules [7], [8]. A closer examination of mitoses that occur in the presence of MMS or the resulting interphase nuclei in cleavage stage Hydractinia embryos revealed surprisingly few problems. Mitotic spindles show robust asters (Figure 2B, B'), different from the case in Drosophila. Relatively normal appearance of cell division could be because 0.1% MMS, though it effectively blocked mitotic activity in the gastrula, does not cause enough damage in cleavage stage nuclei. Alternatively, cleavage stage nuclei may be overcoming the damage sufficiently to continue dividing, albeit more slowly.

Depleting gene products needed for DNA damage detection and repair and assaying for consequences would be an ideal way to address the above-mentioned possibilities. Since such functions remain to be identified in Hydractinia, I resorted to chemical inhibition of DNA damage responses. Caffeine inhibits members of the PI3 Kinase family such as ATM/ATR and is used routinely to inhibit DNA damage and replication checkpoints in fungi, sea urchin embryos and vertebrate cells [e.g.; [20], [21], [22], [23]]. Caffeine at similar, mM, concentrations also inhibit vesicle fusion and cytokinesis in plant cells [e.g.[24]]. I find that 10 mM caffeine on its own inhibited nuclear divisions and cytokinesis in Hydractinia (Table 1 and data not shown). 5 mM caffeine, the lowest amount used in checkpoint studies in animal cells, however, was less disruptive on its own although not completely inert; average nuclear doubling time in caffeine was 32.0 min compared to 21.6 min in controls (Table 1, expt. 5 and 6). Simultaneous treatment of embryos with both 5 mM caffeine and 0.1% MMS produced doubling times that were similar to doubling times in each drug alone (∼30 min; Table 1; expt. 5 and 6). The presence of caffeine in addition to MMS also produced mitotic problems, including the failure to fully separate chromosomes in mitosis (Figure 2C, C'), that were more severe than what is seen in each drug alone (Figure 2 B and D). These results may be interpreted to support the idea that caffeine interferes with normal cellular responses to MMS (see Discussion).

### Survival after MMS exposure is greater in older embryos

To investigate possible developmental changes in resistance to killing by MMS, I quantified the survival of MMS treated embryos. In these experiments, embryos were incubated in 0.1% MMS for 1 hr, followed by transfer to fresh filtered seawater without MMS and further incubation for 24 hr. Typical results from these experiments are shown in Figure 3. Of embryos exposed to MMS at the 4-cell stage in two different experiments, only 5.5% (2/36) survived, in contrast to 100% survival in controls (n = 32). The differences are significant (p<0.001, Fisher's Exact Test). Of embryos exposed to MMS as gastrula (15–16 hr after spawning) in two different experiments, 87% (27/31) survived whereas 100% of controls survived (n = 51). The difference in MMS survival between 4-cell (2/36) and gastrula (27/31) embryos is also significant (p<0.001, Fisher's Exact Test). These results indicate that resistance to killing by MMS is greater in older embryos.

## Discussion

These studies, the first of their kind in embryos of the phylum Cnidaria, show that MMS treatment inhibits mitosis in Hydractinia embryos and that the inhibition is more robust in 12–16 hr old embryos than in 2 and 4-cell embryos. The older embryos are also more resistant to killing by a brief exposure to MMS. Mitosis and chromosome segregation can proceed successfully, if more slowly, in the presence of MMS at concentrations used.

I chose not to use 1-cell embryos in these experiments because comparable concentrations (10 mM) of MMS have been shown to inhibit translation in sea urchin embryos [25], [26], and translation of maternal mRNAs is required to initiate cleavage in many organisms. In fact, the ability of MMS to block the first cleavage in sea urchin could be due not so much to checkpoints but to the failure to synthesize proteins needed for cleavage. Embryos that have successfully completed the first or the second cleavage, in contrast, presumably have completed all translation necessary to initiate cleavage cycles. Thus the effect of MMS on cell division via translation may be minimal at these stages.

Simultaneous treatment of embryos with both 5 mM caffeine and 0.1% MMS produced doubling times that were similar to doubling times in each drug alone (∼30 min; Table 1; expt. 5 and 6). The presence of caffeine in addition to MMS also produced mitotic problems, including the failure to fully separate chromosomes in mitosis (Figure 2C, C'), that were more severe than what is seen in each drug alone. Given the known role of caffeine in inhibition of DNA damage responses, I interpret these data to mean that normal responses to MMS have been compromised by caffeine. In other words, nuclear division delays and relatively normal mitoses seen in the presence of MMS alone no longer occurred when caffeine was also present. This interpretation supports the idea that 0.1% MMS is causing damage but that cells overcome it sufficiently to delay mitosis and cleavage and to avoid gross mitotic abnormalities. The presence of 5 mM caffeine then abrogated responses to MMS and revealed mitotic chromosome segregation problems. Thus, unlike Xenopus, Hydractinia responds to a DNA damaging agent by slowing the entry into mitosis in as early as the 2^nd^ and 3^rd^ cleavage cycle. This behavior is similar to weak checkpoints seen in Drosophila cleavage cycles, although one key difference is that there is no evidence of centrosome inactivation in Hydractinia.

Although Hydractinia cleavage stage embryos respond to MMS by slowing down divisions, inhibition of mitosis is not as robust as in the gastrula (3-fold reduction vs. 15-fold reduction). In other words, cell cycle regulation in response to MMS becomes stronger as the embryo ages. Resistance to killing by MMS exposure is also greater in older embryos. These two features, better cell cycle regulation and increased resistance to a DNA damaging agent after the onset of gastrulation, are shared by Drosophila and Xenopus embryos.

In conclusion, responses to MMS in Hydractinia embryos show similarities to as well as differences from DNA damage responses in Drosophila and Xenopus embryos. Unlike in Xenopus but like in Drosophila, even cleavage cycles slow down in response to MMS in Hydractinia. Unlike in Drosophila, centrosome inactivation is not a response to DNA damage. Similar to the case in Xenopus and Drosophila, cell cycle regulation in the presence of DNA damaging agents as well as resistance to killing by a DNA damaging agent become more robust after the onset of gastrulation in Hydractinia. It would be interesting to investigate whether genotoxins besides MMS can elicit a cell cycle response in Hydractinia cleavage embryos. More important would be to investigate the conservation of DNA damage responses at the molecular level, which would require tools such as antibodies to checkpoint kinases and cell cycle functions in Hydractinia. I hope that this study will encourage other investigators to explore checkpoints and cell cycle regulation in non-traditional experimental systems and to develop tools to take these studies to the molecular level.

## Supporting Information

Figure S1 Major metazoan groupings. Adapted from Halanych KM, Passamaneck Y. 2001. A Brief Review of Metazoan Phylogeny and Future Prospects in Hox-Research. American Zoologist 41: 629-639.
(0.05 MB DOC)

Figure S2 The detection of mitosis in Hydractinia. Cleavage stage embryos were fixed and stained with a DNA dye and with antibodies to phosphorylated Histone H3 (pH3) and β-tubulin. Interphase (inter) and different phases of mitosis are shown. Mitotic stages can be clearly distinguished.
(1.23 MB DOC)
