# Supplementary material for: The Effect of a DNA Damaging Agent on Embryonic Cell Cycles of the Cnidarian Hydractinia echinata
Source: PLoS One. 2010 Jul 23;5(7):e11760. doi: 10.1371/journal.pone.0011760 (PMC2909257; doi:10.1371/journal.pone.0011760)
Supplement: Supplementary file 2 [file pone.0011760.s002.doc]

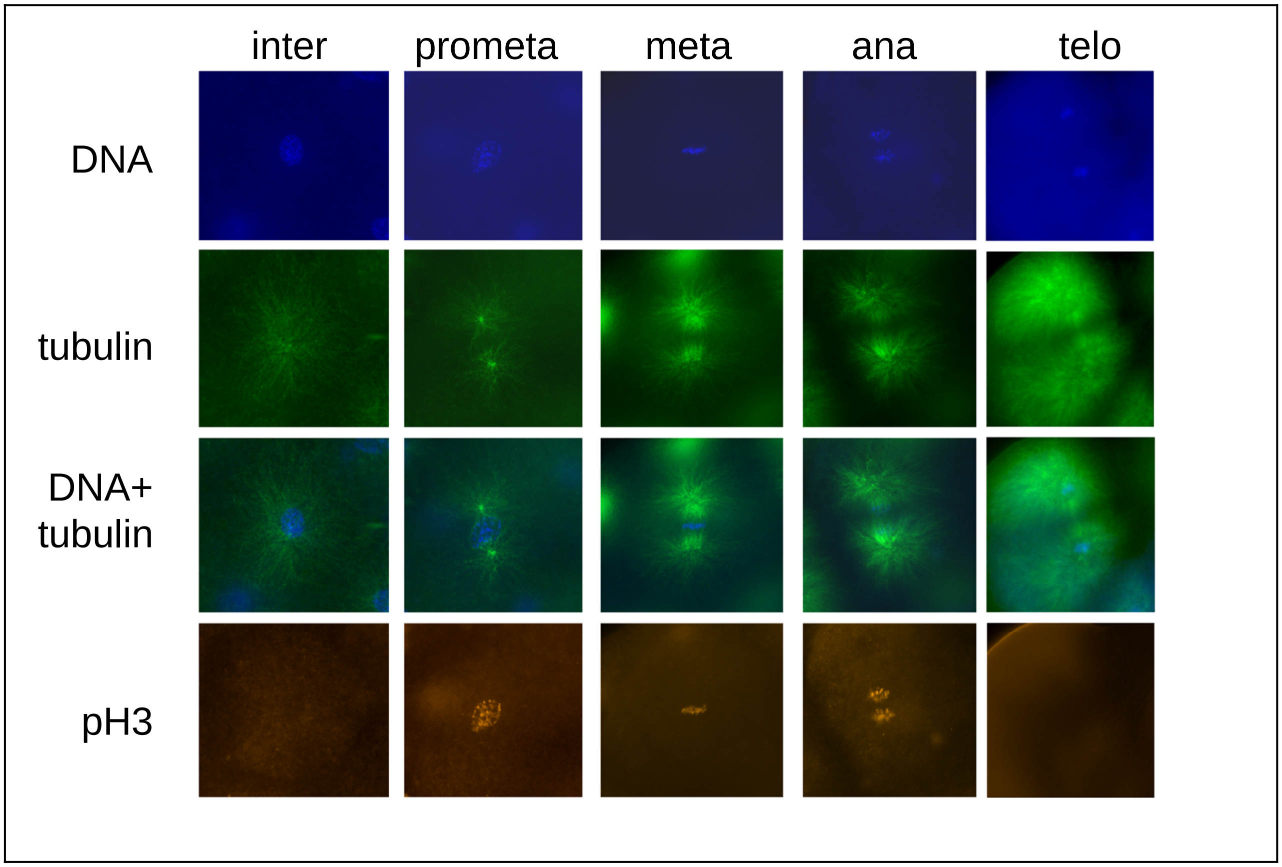

inter
prometa
meta
ana
telo
DNA
tubulin
DNA+ tubulin
pH3
